# Cerebral Ischemic Lesions after Transcatheter Aortic Valve Implantation in Patients with Non-Calcific Aortic Stenosis

**DOI:** 10.3390/jcm11216502

**Published:** 2022-11-02

**Authors:** Xianbao Liu, Dao Zhou, Jiaqi Fan, Hanyi Dai, Gangjie Zhu, Jun Chen, Yuchao Guo, Abuduwufuer Yidilisi, Qifeng Zhu, Yuxin He, Yanxia Wei, Qiong Liu, Xinrui Qi, Jian’an Wang

**Affiliations:** Department of Cardiology, Second Affiliated Hospital Zhejiang University School of Medicine, Hangzhou 310009, China

**Keywords:** non-calcific aortic stenosis, cerebral ischemic lesions, diffusion-weighted magnetic resonance imaging, transcatheter aortic valve implantation

## Abstract

Evidence for transcatheter aortic valve implantation (TAVI) is scarce among patients with non-calcific aortic stenosis, and it is not known whether aortic valve calcification is associated with new cerebral ischemic lesions (CILs) that are detected by diffusion-weighted magnetic resonance imaging. So, our study enrolled 328 patients who underwent transfemoral TAVI using a self-expanding valve between December 2016 and June 2021 from the TORCH registry (NCT02803294). A total of 34 patients were finally confirmed as non-calcific AS and the remaining 294 patients were included in the calcific AS group. Incidence of new CILs (70.6% vs. 85.7%, *p* = 0.022), number of lesions (2.0 vs. 3.0, *p* = 0.010), and lesions volume (105.0 mm^3^ vs. 200.0 mm^3^, *p* = 0.047) was significantly lower in the non-calcific AS group. However, the maximum and average lesion volumes were comparable between two groups. Non-calcific AS was associated with lower risk for developing new CILs by univariate logistic regression analysis [Odds ratio (OR): 0.040, 95% confident interval (CI): 0.18–0.90, *p* = 0.026] and multivariate analysis (OR: 0.031, 95% CI: 0.13–0.76, *p* = 0.010). In summary, non-calcific AS patients had a lower risk of developing new cerebral ischemic infarction after TAVI compared to calcific AS patients. However, new ischemic lesions were still found in over 70% of patients.

## 1. Introduction

Stroke is a devastating complication after transcatheter aortic valve implantation (TAVI). Although the rate of clinical strokes is under 2% among low risk patients, new cerebral ischemic lesions (CILs), which are detected by diffusion-weighted magnetic resonance imaging (DW-MRI), could be found in more than 70% of patients [1]. Recent studies have highlighted the association between new CILs and cognitive impairment [1,2]. The definition of covert stroke was also added in the Valve Academic Research Consortium-3 (VARC-3) [3]. Against a background in which the effectiveness of a cerebral protection device (CPD) was being explored in many randomized controlled studies, new CILs were gradually getting better recognized by clinicians.

Calcific aortic valve stenosis is the most common type of severe aortic stenosis (AS). However, a considerable number of severe AS patients have no or trivial calcification deposition on aortic valves. Cases with non-calcific AS were initially thought to have more difficulties in prosthesis anchoring during TAVI and were excluded in some randomized clinical trials such as PARTNER series studies [4]. Relevant data are limited, although a previous study has suggested the safety and efficacy of TAVI in this population [5]. As brain injury during TAVI may be related to operation in the aortic valve region and it is not well known whether aortic valve calcification is associated with new CILs [6], this study was performed to assess new brain infarcts detected by DW-MRI in patients with non-calcific AS.

## 2. Materials and Methods

The study consecutively enrolled 328 patients who underwent transfemoral TAVI using self-expanding valves between December 2016 and June 2021 from the TORCH (Transcatheter Aortic Valve Replacement Single Center Registry in Chinese Population, a prospective cohort study; NCT02803294) registry. Exclusion criteria were: (1) underwent valve-in-valve TAVI; (2) a stroke or transient ischemic attack within six months before TAVI; (3) unplanned cardiopulmonary bypass or conversion to open surgery during a procedure; (4) contraindication for MRI before or after TAVI (such as implantation of an incompatible pacemaker or a metallic foreign body); (5) inability to tolerate MRI examination due to a clinical situation; (6) poor image quality or outside of the appropriate time frame (>7 days); (7) absence of MRI or pre-operative computed tomography (CT) examination taken for other reasons. The study was approved by the ethics committee of the Second Affiliated Hospital of Zhejiang University, and written informed consent was obtained from all participants.

**Figure 1:**
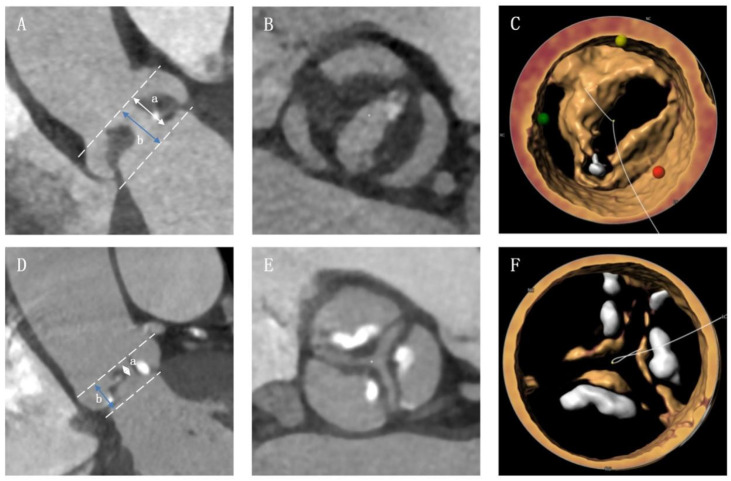
Typical image of non-calcific and calcific aortic valve stenosis. (**A**) The non-calcific AS patient should have a a/b ≥ 50% on coronal oblique view; (**B**) non-calcific AS patient had leaflets with continuous and diffuse low-density thickening on axial reconstruction; (**C**) patient was confirmed as non-calcific AS in 3D-volume rendering reconstruction. (**D**–**F**) images of calcific AS.

All patients underwent a standard electrocardiograph-gated contrast-enhanced multi-slice computed tomography (MSCT) before TAVI procedure. Best systolic phase was used to evaluate aortic root anatomy using 3mensio software (Bilthoven, the Netherlands). The severity of valve calcification was first classified semi-quantitatively into none (grade I), mild (grade II), moderate (grade III) and severe, as described previously [7]. Then, valve calcific severity was categorized as either heavy (grade IV) or massive (grade V) in patients with severe valve calcification [8]. Non-calcific AS was defined as severe aortic valve stenosis with minimal calcification deposition and obvious leaflet thickening [5]. The pre-operative CT images of patients with grade I or II aortic valve calcification were screened, and non-calcific AS was identified by two independent authors (DZ and JQF) based on the standard described in a previous study [5]. Patients who fulfilled all the following four criteria were enrolled in non-calcific AS group: (1) all leaflets had continuous and diffuse low-density thickening on axial reconstruction; (2) ratio of the height of hypoattenuating opacities to the distance between the annulus and the tip of valve ≥ 50% on coronal oblique view; (3) there was obvious leaflet thickening and no/trivial calcium deposition as confirmed by 3D-volume rendering reconstruction; and (4) leaflet thickening was reported in pre-operative echocardiography. Typical image of non-calcific AS and calcific-AS were provided in Figure 1. The device landing zone calcium volume score was also evaluated. Usually, the threshold for detecting calcification was set at 850 Hounsfield units (HU). If the contrast was too great, an optimized threshold was used based on 3mensio software automatic generation or mean HU + 100 HU (the regions of interest were placed in the ascending aorta or in the left ventricle to determine mean HU). The bicuspid or tricuspid aortic valve was identified in several phases of pre-operative contrast enhanced MSCT by two experienced cardiologists (JQF and QFZ) and was confirmed by two authors (DZ and YCG) according to the Sievers’s classification [9]. The oversizing ratio was calculated using the following formula: *oversizing by perimeter (%) = (prosthesis inflow nominal perimeter/measured perimeter − 1) × 100%* [10].

The TAVI for patients was decided by our multidisciplinary heart team. In our center, self-expanding TAVI was performed most frequently, and transfemoral access was preferred if there were no contraindications. To reduce bias, this study only enrolled patients who underwent transfemoral TAVI with a self-expanding valve such as VenusA valve (Venus Medtech), Taurusone valve (Peijia Medical), ProStyle valve (KingstronBio), or Vitaflow (Microport) and their series. Heparin was used in all procedures (50–70 U/kg), which was adjusted by activating clotting time (>250 s). Patients routinely received pre-dilatation, and post-dilatation was decided by operators based on intraoperative situation. Hangzhou Solution procedural strategy was used to decide prosthesis size selection in bicuspid aortic valve stenotic patients [10,11]. Other details of procedural strategy can be found in our previous studies [12,13]. The clinical adverse events were defined following the Valve Academic Research Consortium-2 consensus [14]. Patients were routinely required to undergo echocardiography before and after TAVI, and at the 30-day follow up.

In this study, the brain DW-MRI was performed in all patients before TAVI, and within seven days after the procedure. The imaging was acquired following our standardized scan protocol in a 1.5-T or a 3.0-T system, as described in our previous study [12]. The post-operative MRI image was compared to the baseline image to detect new CILs. The new cerebral ischemic lesions were evaluated in DWI sequence, fluid-attenuated inversion recovery sequence, and apparent diffusion coefficient maps by two independent authors, and they were confirmed by a neurologist. The analysts only knew that the patients underwent TAVI and their basic information such as the patient’s name and gender, without knowing the patient’s anatomical data (including echocardiographic and CT characteristics) and procedural data [15]. The volume of CILs was analyzed in DWI sequence using MRIcron software Version 4 (NeuroImaging Tools and Resources Collaboratory, South Carolina). The location of new lesions and vascular territories were classified as per previous studies, including the anterior cerebral artery, middle cerebral artery, posterior cerebral artery, vertebral artery, and basilar artery [12,16,17].

The continuous variables were presented as mean ± standard deviation or median (interquartile range, IQR) and were compared by Unpaired Student-t test or Mann-Whitney U test according to the distribution as determined by Shapiro-Wilk test. All categorical data were expressed as numbers (percentages) and were compared using χ^2^ or Fisher exact test. Univariate logistic regression analysis was performed to evaluate potential predictors of developing new CILs after TAVI. The variables with a *p*-value < 0.20 in univariate analysis were included in multivariate logistic regression analysis using a backward likelihood ratio method. In addition, receiver operating characteristic analysis was performed and the Youden index was calculated to determine the optimal cut-off for important continuous variables. A two-tail *p*-value of <0.05 was considered statistically significant. The SPSS software version 20.0 (IBM) was used for statistical analyses.

## 3. Results

Among 54 patients with no or mild calcification who were screened for leaflet thickening and valve calcification, a total of 34 patients were finally confirmed as non-calcific AS, and the remaining 294 patients were included in the control group. Baseline characteristics are shown in Table 1. There was no statistical difference between the two groups in age (72.0 years vs. 74.0 years, *p* = 0.102) or in the Society of Thoracic Surgeons score (3.92 vs. 4.05, *p* = 0.625). The body surface area was lower (1.55 kg/m^2^ vs. 1.63 kg/m^2^, *p* = 0.001) whereas more females (82.4% vs. 39.5%, *p* < 0.001), more patients with diabetes (38.2% vs. 20.4%, *p* = 0.018) and more patients with cancer history (8.8% vs. 1.7%, *p* = 0.050) were found in the non-calcific AS group. Additionally, fewer patients suffered the New York Heart Association (NYHA) heart function of class III/IV symptoms, and a significantly higher left ventricular ejection fraction was found in the non-calcific group. In the analyses of MSCT data, fewer bicuspid AS (35.3% vs. 64.6%, *p* = 0.001) and smaller aortic root anatomies could be found in this population. There was no other baseline characteristic difference between the two groups, which included hypertension, stroke history, atrial fibrillation or flutter, etc. Antithrombotic therapy was also comparable between the two groups (Table S1).

The VenusA series prosthesis was most frequently (79.9%) used among all self-expanding valves. Pre-dilatation was performed in all but one patient in the entire population (Table 2). The oversizing ratio calculated by annular perimeter was significantly larger (12.10% vs. 6.84%, *p* < 0.001) in the non-calcific AS group. These two populations had similar risks for second valve implantation, with 5.9% and 8.8% rates in this study, respectively. The clinical stroke rates were comparable between patients with non-calcific and calcific AS during the 30-days follow-up (Table S2). No patient suffered from an overt stroke in the non-calcific AS group, whereas 3.1% of patients in the calcific AS group underwent a clinical stroke. Other clinical events such as mortality, bleeding, pacemaker implantation and new-onset atrial fibrillation were also comparable between two groups. Although the moderate or severe paravalvular leakage rate was similar, there were lesser patients with mild or more paravalvular leakage in the non-calcific group (26.5% vs. 63.9%, *p* < 0.001) before discharge. Similar results were also found in the 30-day follow-up echocardiography (Table S1).

**Figure 2:**
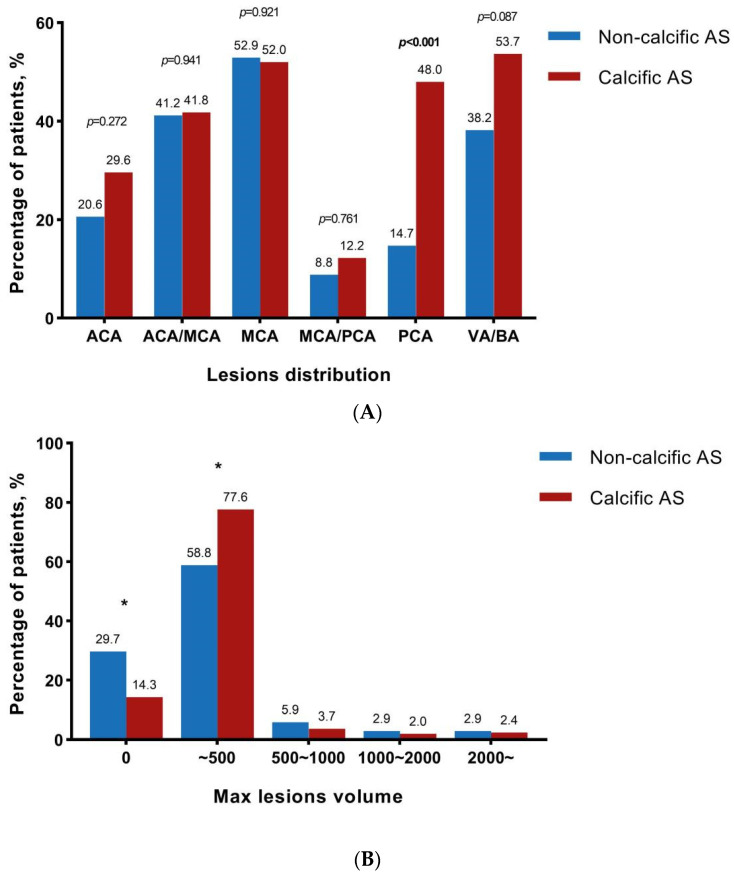
CILs distribution and maximum lesions volume. * represented statistically significant. (**A**) The percentage of patients who had new CILs in different vascular territories including anterior cerebral artery (ACA), middle cerebral artery (MCA), posterior cerebral artery (PCA), vertebral artery, and basilar artery (VB/BA), ACA/MCA and MCA/PCA; (**B**) The percentage of patients who had a maximum volume of CILs lower than 500 mm^3^, 500~1000 mm^3^, 1000~2000 mm^3^, or ≥2000 mm^3^.

The information on new cerebral ischemic lesions is provided in Table 3 and Figure 2. The post-operative DW-MRI was performed at a similar number of days after TAVI between the two groups [3.0 days (IQR: 1.0–5.0 days) vs. 2.5 days (IQR: 1.0–5.0 days), *p* = 0.910]. The incidence of new CILs was significantly lower in the non-calcific AS group (*p* = 0.022), whereas there 70.6% of patients in this population still developed new CILs. Compared to calcific AS patients, non-calcific AS patients had a lower risk for CILs not only with respect to the number of lesions [2.0 (IQR: 0–4.0) vs. 3.0 (IQR: 1.0–7.3), *p* = 0.010], but also for the total volume of lesions [105.0 (IQR: 0–332.5) mm^3^ vs. 200.0 (IQR: 70.0–570.0) mm^3^, *p* = 0.047]. However, the maximum lesion volume and the average lesion volume were found to be comparable between the two groups. While considering the distribution of lesion location, fewer patients with non-calcific AS had new CILs in posterior cerebral artery zones (14.7% vs. 48.0%, *p* < 0.001, Figure 2A). No significant difference was found between the two groups in other cerebral artery zones. Further analysis of maximum lesion volume showed that fewer patients had a maximum lesion volume lower than 500 mm^3^ (Figure 2B). However, the number of patients with a maximum lesion volume of 500~1000 mm^3^, 1000~2000 mm^3^, or ≥2000 mm^3^ was similar between the two groups (Figure 2B).

Patients were divided into a non-calcific AS group and a calcific AS group. Non-calcific AS patients had a lower incidence of new CILs, fewer lesions, and lower total volume of lesions. Non-calcific AS and moderate or severe mitral regurgitation (MR) were independent protective factors of new CILs after TAVI, while higher aortic valve peak velocity and larger oversizing ratio by annulus perimeter were independent risk factors for new CILs. 

The results of the logistic regression analysis are shown in Table 4 and Table S3. Non-calcific AS was associated with lower risk of new CILs both in univariate logistic regression analysis [Odds ratio (OR): 0.040, 95% confident interval (CI): 0.18–0.90, *p* = 0.026] and multivariate analysis (OR: 0.031, 95% CI: 0.13–0.76, *p* = 0.010). Besides, the max velocity of ≥5 m/s and oversizing ratio by annulus perimeter of ≥6.90% were independent risk factors, whereas moderate/severe mitral regurgitation was an independent protective factor for new CILs. In another regression analysis model that included the severity of valve calcification a more severe degree of valve calcification was an independent risk factor of new CILs in a multivariate logistic regression analysis (OR: 1.47, 95% CI: 1.02–2.11, *p* = 0.037, Table S4). In addition, a high calcium score volume (cut-off determined by the YOUDEN index) was associated with a higher risk of new CILs in both univariate and multivariate logistic regression analyses [univariate: OR: 1.85 (1.01–3.39), *p* = 0.047; multivariate: OR: 2.30 (1.19–4.43), *p* = 0.013, Table S5].

## 4. Discussion

The present study is the first to evaluate post-operative cerebral ischemic lesions in the non-calcific AS population. The main findings were: (1) patients with non-calcific AS had less brain injury, including the number and volume of lesions, compared to those with calcific AS; (2) although the incidence of post-operative lesions was lower in the non-calcific AS population, there were still over 70% of patients who developed new CILs after TAVI; (3) a more severe aortic valve calcification based on the MSCT evaluation was an independent predictor for new CILs; and (4) a higher aortic peak flow velocity before TAVI, without moderate or severe MR, and a larger oversizing ratio were independent predictors of the new-onset post-operative CILs.

Since the indications of TAVI have been expanded according to the updated guideline, TAVI’s complications should be understood more deeply [18,19,20]. Although some studies demonstrated that patients who underwent TAVI had a similar or a lower incidence of overt stroke compared to those who underwent surgical aortic valve replacement (SAVR) [21,22], it has also been proved that the DW-MRI commonly detected CILs after TAVI, and patients who underwent TAVI often had more lesions compared to those who underwent SAVR [17,23]. In addition, recent evidence suggested that covert CILs were not silent, and could impair cognitive functions [1,2]. Therefore, covert brain injury after TAVI is receiving more attention currently.

Although calcific AS was the main reason behind severe AS in most patients, other mechanisms can lead to its development. Pathological changes in non-calcific AS are diverse and include systemic lupus erythematosus, rheumatic heart disease, and early stage calcific AS [5]. More female patients, more patients with diabetes mellitus, and more patients with cancer history in non-calcific AS group also suggest the different characteristics of non-calcific AS patients. The non-calcific AS population in those undergoing TAVI was first identified in a study by Xiong TY et al. [5]. They illustrated that this was a relative common cause of severe AS and the proportion of non-calcific AS patients was 15.4% in their study. Likewise, the proportion of non-calcific AS patients was 10.3% in our study, revealing that this was a non-negligible population. Since non-calcific AS patients were not well studied, and the population characteristics were special, it was valuable to evaluate brain injury after TAVI in this population.

There exist knowledge gap in the association between CILs and aortic valve calcification. The first study to evaluate the etiopathology of the embolized material was carried out in 2013, which reported that debris captured by the filter-based CPD contained calcium [24]. Nevertheless, calcified materials could only be found in 17% of all TAVI patients in this study. Similar low incidences of captured calcified debris (less than 30% of TAVI patients) were also found in Van Mieghem et al. and Kroon et al.’s studies [25,26]. In fact, capturing calcified materials did not mean that aortic valve calcification was an independent predictor of CILs, whereas aortic wall and thrombus were found as debris most frequently [25,26,27]. A study by Aratake et al. suggested the Agatston score of aortic valve was associated with larger amounts of high-intensity transient signals (HITS), which represented micro-embolization and were assessed by monitoring blood flow in the cervical arteries [28]. However, there was insufficient evidence to prove the association between CILs in MRI imaging and HITS. Besides, although previous research suggested that aortic valve calcification assessed by echocardiography related to a higher risk of new CILs, this finding has not been proved in any CTA analyses [16]. Evaluating calcification scores in echocardiography could be easily interfered with by thickening tissue, which is another important pathology that leads to AS. In our study, calcific AS patients had more severe CILs that were detected by DW-MRIs both in the number of lesions and their volumes, revealing a higher risk for developing CILs in those with calcific AS. Moreover, regression analysis of new CILs, which contain aortic valve calcification severity or aortic valve calcium volume score, also suggested aortic valve calcification was an independent predictor of new CILs.

Some CPDs were believed to reduce CILs after TAVI and were thought to reduce clinical stroke in some real-world database studies [29,30]. However, the effectiveness of CPD to protect patients from overt stroke has not been proved by any randomized controlled studies. Applications of CPDs was a hot topic and it remains unknown which kinds of patients should receive CPD during TAVI. In fact, the influential factors of brain injury were highly heterogeneous. According to current knowledge, both patient and operative factors could affect brain injury after TAVI. Notably, our study illustrated that 70% of patients developed CILs in the non-calcific AS group even if the number and volume of CILs were lower compared to calcific AS group. Besides, large lesions, which had more chances of causing a neurological deficit, were comparable between non-calcific and calcific AS patients in our study. This suggested that post-operative CILs were non-negligible in the non-calcific AS population. Thus, CPD could also be considered in this population, especially in the further exploration of its efficacy.

Our multivariate regression analysis model saw a larger oversizing ratio and higher aortic peak velocity before TAVI as independent risk factors for the development of new CILs. Increased oversizing ratio meant larger radial force during the prosthesis implantation, which could cause mechanical injury to the artery and the aortic valve, leading to more embolus fragment falling off and cerebral embolization. In our center, the application of Hangzhou Solution can decrease the oversizing ratio in bicuspid aortic valve patients, who are at high risk of brain injury [12]. In a study that enrolled 2621 patients from the PARTNER trial, higher aortic peak velocity was an independent risk factor for early stroke after transfemoral-TAVI [31]. Samim et al. also found that higher aortic peak velocity was independently associated with a larger total volume of post-operative CILs [32]. In line with other studies, our study found that the max aortic velocity ≥ 5 m/s was also confirmed as an independent risk factor of new CILs. On the one hand, higher aortic peak velocity meant more severe AS. More thickening tissue and calcification may lead to a higher chance of an embolus falling off. On the other hand, higher blood flow velocity was more likely to shock off the embolus fragment, and a stronger, forward blood flow could transport the embolus to the cerebral artery. In addition, we also found that moderate or severe mitral valve regurgitation was an independent protective factor against CILs. This could be due to the backward blood flow in the mitral valve reducing the high-velocity jet impact in the aortic valve region during the systolic phase. Nevertheless, the actual mechanism needs to be further explored in future research.

## 5. Limitation

Our study has limitations. First, there were only 34 patients in the non-calcific group. The result that non-calcific AS patients had less brain injury needed to be further verified given the small sample size in this study. Secondly, since most of the prostheses used in our center are self-expanding valves, we only included patients using self-expanding valves in this study. This meant that there was selection bias, and the result should not be generalized to patients using balloon-expandable valves or patients using mechanically expandable valves. Additionally, although data were collected in our prospective registry, and our imaging protocol was developed before trial registration, there were still many patients who could not be included in the study for different reasons. For example, many patients with incompatible pacemaker implantation were excluded, making the rate of prior pacemaker and permanent pacemaker implantation after TAVI quite low. In addition, patients in the worst clinical situations, such as patients converted to open surgery or patients intolerant to MRI examination, were excluded, resulting in the “perfect” short-term clinical outcomes. Therefore, clinical results in this study could not completely represent outcomes of the whole non-calcific and calcific AS population. Future large-scale and randomized studies are needed to confirm the result.

## 6. Conclusions

Compared to patients with calcific AS, patients with non-calcific AS had a significantly lower incidence of developing new cerebral ischemic infarction, fewer lesions, and lower total lesions volume after TAVI. However, new ischemic lesions were still found in over 70% of patients, and the volume of the largest lesion was similar between the two groups. The risk of developing CILs in this population after TAVI should be more deeply understood by clinicians.

## Figures and Tables

**Table 1:** Baseline Characteristics.

	Non-Calcific AS *n* = 34	Calcific AS *n* = 294	*p* Value
Ages, yrs	72.0 (69.0–76.0)	74.0 (69.0–79.3)	0.102
Female	28 (82.4%)	116 (39.5%)	**<0.001**
Body mass index, kg/m^2^	22.89 ± 3.24	22.93 ± 3.41	0.955
Body surface area, m^2^	1.55 ± 0.12	1.63 ± 0.16	**0.001**
STS score, %	3.92 (2.41–5.89)	4.05 (2.43–7.23)	0.625
Smoker	4 (11.8%)	65 (22.1%)	0.161
Hypertension	22 (64.7%)	161 (54.8%)	0.269
Diabetes mellitus	13 (38.2%)	60 (20.4%)	**0.018**
Chronic kidney disease stage 4 or 5	2 (5.9%)	26 (8.8%)	0.794
History of cancer	3 (8.8%)	5 (1.7%)	**0.050**
NYHA class III/IV	21 (61.8%)	241 (82.0%)	**0.005**
Atrial fibrillation/flutter	5 (14.7%)	47 (16.0%)	0.847
Prior myocardial infarction	1 (2.9%)	2 (0.7%)	0.281
Prior PCI	2 (5.9%)	30 (10.2%)	0.618
Prior stroke	1 (2.9%)	10 (3.4%)	1.000
Prior pacemaker implantation	1 (2.9%)	0 (0%)	0.104
Chronic obstructive pulmonary disease	8 (23.5%)	66 (22.4%)	0.887
Echocardiographic data			
LVEF, %	63.6 (58.6–68.4)	59.2 (46.5–64.0)	**0.001**
Max velocity, m/s	4.59 (4.22–5.01)	4.78 (4.33–5.46)	**0.044**
Mean gradient, mmHg	46.5 (40.0–57.3)	53.0 (43.0–70.0)	**0.015**
Aortic valve area, cm^2^	0.70 (0.50–0.81)	0.58 (0.45–0.75)	0.213
≥moderate aortic regurgitation	17 (50.0%)	117 (39.8%)	0.252
≥moderate mitral regurgitation	10 (29.4%)	73 (25.0%)	0.576
≥moderate tricuspid regurgitation	4 (11.8%)	37 (12.7%)	1.000
Computed tomography data			
Calcium volume score	83.0 (26.0–183.8)	572.5 (308.8–1007.4)	**<0.001**
Bicuspid aortic valve	12 (35.3%)	190 (64.6%)	**0.001**
Max diameter, mm	25.0 (23.9–26.8)	27.5 (25.8–29.5)	**<0.001**
Min diameter, mm	19.9 (18.2–21.8)	21.3 (19.9–23.2)	**<0.001**
Perimeter, mm	71.3 (66.1–76.0)	77.3 (72.8–83.1)	**<0.001**
Perimeter derived diameter, mm	22.7 (21.1–24.2)	24.6 (23.2–26.4)	**<0.001**
Area, mm^2^	392.5 (337.7–447.1)	454.0 (405.4–526.5)	**<0.001**
STJ diameter, mm	28.7 ± 4.8	30.6 ± 4.2	**0.015**
Ascent aorta diameter at 4 cm, mm	37.1 ± 5.7	37.9 ± 4.5	0.301
Right coronary artery height, mm	16.0 (13.6–18.1)	16.7 (14.7–18.6)	0.107
Left coronary artery height, mm	12.9 (11.6–14.3)	14.7 (12.6–17.0)	**<0.001**
Aortic root angle, degree	50.0 ± 10.3	51.2 ± 10.5	0.512

Data was presented as *n* (%) or mean ± SD or median (interquartile range, IQR). *p* Values in bold are statistically significant. LVEF = left ventricular ejection fraction; NYHA = New York Heart Association; PCI = percutaneous coronary intervention; STJ = sinotubular junction; STS score = Society of Thoracic Surgeons score.

**Table 2:** Procedural Characteristics and Peri-operative Outcomes.

	Non-Calcific AS *n* = 34	Calcific AS *n* = 294	*p* Value
Pre-dilatation	34(100%)	293(99.7%)	1.000
Post-dilatation	18(52.9%)	201(68.4%)	0.071
Oversizing ratio by perimeter, %	12.10 ± 7.86	6.84 ± 7.92	**<0.001**
Second valve implantation	2(5.9%)	26(8.8%)	0.794
Annular rupture	0(0%)	1(0.3%)	1.000
Coronary obstruction	0(0%)	1(0.3%)	1.000
Echocardiographic data before discharge			
LVEF, %	61.2(57.7–66.6)	60(51.0–65.9)	0.103
Max velocity, m/s	2.45(2.16–2.74)	2.31(1.99–2.67)	0.106
Mean gradient, mmHg	11.0(10.0–16.0)	11.0(8.0–15.0)	0.150
Aortic valve area, cm^2^	1.55(1.16–1.71)	1.57(1.32–1.82)	0.208
≥mild paravalvular leakage	9(26.5%)	188(63.9%)	**<0.001**
≥moderate paravalvular leakage	0(0%)	19(6.5%)	0.254

Data was presented as *n* (%) or mean ± SD or median (interquartile range, IQR). *p* Values in bold are statistically significant. LVEF = left ventricular ejection fraction.

**Table 3:** Comparison of new cerebral ischemic lesions.

	Non-Calcific AS *n* = 34	Calcific AS *n* = 294	*p* Value
Patients with new lesions	24 (70.6%)	252 (85.7%)	**0.022**
New lesions per patient	2.0 (0–4.0)	3.0 (1.0–7.3)	**0.010**
Patients with a single lesion	6 (17.6%)	33 (11.2%)	0.415
Patients with multiple lesions	18 (52.9%)	219 (74.5%)	**0.008**
Total new lesions	130	1590	
Patients with new lesions in different location			
ACA	7 (20.6%)	87 (29.6%)	0.272
ACA/MCA	14 (41.2%)	123 (41.8%)	0.941
MCA	18 (52.9%)	153 (52.0%)	0.921
MCA/PCA	3 (8.8%)	36 (12.2%)	0.761
PCA	5 (14.7%)	141 (48.0%)	**<0.001**
VA/BA	13 (38.2%)	158 (53.7%)	0.087
Maximal lesion volume, mm^3^, per patient	70.0 (0–127.5)	90.0 (40.0–200.0)	0.151
Average lesion volume, mm^3^, per patient	60.0 (0–79.0)	55.0 (29.5–90.0)	0.461
Total lesion volume, mm^3^, per patient	105.0 (0–332.5)	200.0 (70.0–570.0)	**0.047**
MRI time after procedure, days	3.0 (1.0–5.0)	2.5 (1.0–5.0)	0.910

Data was presented as *n* (%) or median (interquartile range, IQR). *p* Values in bold are statistically significant. ACA = anterior cerebral artery; AS = aortic stenosis; BA = basilar artery; DW-MRI = diffusion-weighted magnetic resonance imaging; MCA = middle cerebral artery; PCA = posterior cerebral artery; TAVI = transcatheter aortic valve implantation; VA = vertebral artery.

**Table 4:** Univariate and multivariate logistic regression analysis of new CILs.

	Univariate Regression		Multivariate Regression	
	*p* Value	OR (95% CI)	*p* Value	OR (95% CI)
Non-Calcific AS	**0.026**	0.40(0.18–0.90)	**0.010**	0.31(0.13–0.76)
Bicuspid aortic stenosis	0.063	1.76(0.97–3.20)	-	-
Diabetes mellitus	0.110	0.59(0.30–1.13)	-	-
Dyslipidemia	0.135	2.53(0.75–8.53)	-	-
Max velocity ≥ 5 m/s	0.070	1.82(0.95–3.46)	**0.048**	1.97(1.01–3.86)
Pure AS *	**0.050**	3.35(1.00–11.21)	0.075	3.06(0.89–10.51)
Moderate/severe MR	**0.008**	0.43(0.23–0.80)	**0.026**	0.48(0.25–0.92)
Oversizingratio by annulus perimeter > 6.90%	0.105	1.64(0.90–2.98)	**0.025**	2.12(1.10–4.09)
MRI time	0.078	0.88(0.76–1.01)	-	-

The variables with a *p* value < 0.20 in univariate analysis were included in a multivariate logistic regression analysis using a backward likelihood ratio method. No multicollinearity existed among the variables in multivariate regression model. ROC curve analysis was performed for important continuous variables and the optimal cut-off was determined using the Youden Index. *p* Values in bold are statistically significant. * Pure AS represented severe aortic stenosis without mild or more aortic regurgitation; AS = aortic stenosis; MR = mitral regurgitation.

## Data Availability

The data presented in this study are available on request from the corresponding author. The data are not publicly available due to privacy restrictions.

## Supplementary Materials

The following supporting information can be downloaded at: https://www.mdpi.com/article/10.3390/jcm11216502/s1, Table S1: Antithrombotic therapy before and after TAVI; Table S2: Clinical Outcomes at 30-day Follow up; Table S3: Univariate logistic regression analysis of new CILs; Table S4: Univariate and multivariate logistic regression analysis of new CILs (include valve calcification severity); Table S5: Univariate and multivariate logistic regression analysis of new CILs (Include calcium volume score).
