# Supplementary material for: Cerebral Ischemic Lesions after Transcatheter Aortic Valve Implantation in Patients with Non-Calcific Aortic Stenosis
Source: J Clin Med. 2022 Nov 2;11(21):6502. doi: 10.3390/jcm11216502 (PMC9655232; doi:10.3390/jcm11216502)
Supplement: Supplementary file 1 [file jcm-11-06502-s001.zip › jcm-1948540-supplementary.pdf]

Table S1. Antithrombotic therapy before and after TAVI

	Non-calcific AS<br>n = 34	Calcific AS<br>n = 294	P Value
Medication before TAVI	-	-	0.535
No antithrombotic therapy	7(20.6%)	96(32.7%)	-
Antiplatelet	23(67.6%)	168(57.1%)	-
Anticoagulant	3(8.8%)	20(6.8%)	-
Antiplatelet plus anticoagulant	1(2.9%)	10(3.4%)	-
Medication after TAVI	-	-	0.688
No antithrombotic therapy	0(0%)	1(0.3%)	-
Antiplatelet	26(76.5%)	244(83.0%)	-
Anticoagulant	6(17.6%)	32(10.9%)	-
Antiplatelet plus anticoagulant	2(5.9%)	17(5.8%)	-

TAVI = transcatheter aortic valve implantation

Table S2. Clinical Outcomes at 30-day Follow up

	Non-calcific AS n = 34	Calcific AS n = 294	P Value
All-cause Mortality	0(0%)	1(0.3%)	1.000
Cardiovascular mortality	0(0%)	1(0.3%)	1.000
Myocardial infarction	0(0%)	1(0.3%)	1.000
Stroke	0(0%)	9(3.1%)	0.631
Disabling stroke	0(0%)	3(1.0%)	1.000
Non-disabling stroke	0(0%)	6(2.0%)	0.869
Life-threatening bleeding	0(0%)	1(0.3%)	1.000
New permanent pacemaker implantation	0(0%)	11(3.7%)	0.519
New-onset atrial fibrillation	2(5.9%)	10(3.4%)	0.805
NYHA class III/IV	2(5.9%)	21(7.3%)	1.000
**Echocardiagraphic data**			
LVEF, %	62.4(58.1-66.0)	61.0(55.1-65.0)	0.161
Max velocity, m/s	2.25±0.38	2.30±0.53	0.426
Mean gradient, mmHg	11.0(8.8-13.3)	11.0(7.0-14.0)	0.999
Aortic valve area, cm^2	1.46(1.20-1.72)	1.57(1.35-1.79)	0.086
≥ mild paravalvular leakage	14(41.2%)	178(63.6%)	**0.011**
≥ moderate paravalvular leakage	0(0%)	15(5.4%)	0.338

Data was presented as n (%) or median (interquartile range, IQR). p Values in bold are statistically significant. LVEF = left ventricular ejection fraction; NYHA = New York Heart Association

Table S3. Univariate logistic regression analysis of new CILs.

	Univariate regression	
	P value	OR (95%CI)
Female sex	0.801	1.08(0.59-1.97)
Obesity	0.598	0.65(0.13-3.22)
Previous stroke	0.539	1.92(0.24-15.31)
Prior atrial fibrillation/flutter	0.975	0.99(0.43-2.25)
LVEF, per 1 percent	0.340	1.01(0.99-1.03)
LVEF ≤ 40%	0.993	1.00(0.42-2.38)
Medication before TAVI	0.420	-
No antithrombotic therapy	Ref	Ref
Antiplatelet	0.169	0.61(0.30-1.24)
Anticoagulant	0.475	0.48(0.15-1.51)
Antiplatelet plus anticoagulant	0.800	1.32(0.16-11.23)
Medication after TAVI*	0.148	-
Antiplatelet	Ref	Ref
Anticoagulant	0.128	0.53(0.23-1.20)
Antiplatelet plus anticoagulant	0.156	0.46(0.16-1.35)
Non-calcific AS	**0.026**	0.40(0.18-0.90)
Valve calcification severity , per one degree	0.082	1.34(0.96-1.85)
Calcium volume score ≥494 mm^3	**0.047**	1.85(1.01-3.39)
Bicuspid aortic stenosis	0.063	1.76(0.97-3.20)
Diabetes mellitus	0.110	0.59(0.30-1.13)
Dyslipidemia	0.135	2.53(0.75-8.53)
Max velocity ≥5 m/s	0.070	1.82(0.95-3.46)
Pure AS†	**0.050**	3.35(1.00-11.21)
Moderate/severe MR	**0.008**	0.43(0.23-0.80)
Postdilatation	0.384	1.31(0.71-2.42)
Second valve implantation	0.203	2.60(0.60-11.31)
Oversizing ratio by annulus perimeter ≥6.9%	0.105	1.64(0.90-2.98)
MRI time	0.078	0.88(0.76-1.01)

* Since only 1 patient received no antithrombotic therapy after TAVI for the consideration of bleeding risk, this patient was not included in the analysis of medication after TAVI. † Pure AS represented severe aortic stenosis without more than trace aortic regurgitation; Obesity was defined as BMI > 30 kg/m^2. ROC curve analysis were performed for important continuous variables (calcium volume score, max velocity, oversizing ratio by annulus perimeter) and the optimal cut-off were determined using Youden Index.

AS = aortic stenosis; MR = mitral regurgitation;

Table S4. Univariate and multivariate logistic regression analysis of new CILs (Include valve calcification severity)

	Univariate regression		Multivariate regression	
	P value	OR (95%CI)	P value	OR (95%CI)
Valve calcification severity, per one degree	0.082	1.34(0.96-1.85)	**0.037**	1.47(1.02-2.11)
Bicuspid aortic stenosis	0.063	1.76(0.97-3.20)	-	-
Diabetes mellitus	0.110	0.59(0.30-1.13)	-	-
Dyslipidemia	0.135	2.53(0.75-8.53)	-	-
Max velocity ≥5 m/s	0.070	1.82(0.95-3.46)	0.085	1.81(0.92-3.55)
Pure AS*	**0.050**	3.35(1.00-11.21)	0.076	3.04(0.89-10.39)
Moderate/severe MR	**0.008**	0.43(0.23-0.80)	**0.016**	0.45(0.24-0.86)
Oversizing ratio by annulus perimeter ≥6.9%	0.105	1.64(0.90-2.98)	**0.026**	2.10(1.09-4.02)
MRI time	0.078	0.88(0.76-1.01)	-	-

The variables with a p value <0.20 in univariate analysis were included in multivariate logistic regression analysis using a backward likelihood ratio method. No multicollinearity existed among the variables in multivariate regression model. ROC curve analysis were performed for important continuous variables and the optimal cut-off were determined using Youden Index.

* Pure AS represented severe aortic stenosis without more than trace aortic regurgitation; AS = aortic stenosis; MR = mitral regurgitation;

Table S5. Univariate and multivariate logistic regression analysis of new CILs (Include calcium volume score)

	Univariate regression		Multivariate regression	
	P value	OR (95%CI)	P value	OR (95%CI)
Calcium volume score ≥494 mm^3	**0.047**	1.85(1.01-3.39)	**0.013**	2.30(1.19-4.43)
Bicuspid aortic stenosis	0.063	1.76(0.97-3.20)	-	-
Diabetes mellitus	0.110	0.59(0.30-1.13)	-	-
Dyslipidemia	0.135	2.53(0.75-8.53)	-	-
Max velocity ≥5 m/s	0.070	1.82(0.95-3.46)	-	-
Pure AS*	**0.050**	3.35(1.00-11.21)	0.075	3.06(0.90-10.46)
Moderate/severe MR	**0.008**	0.43(0.23-0.80)	**0.026**	0.48(0.26-0.92)
Oversizing ratio by annulus perimeter ≥6.9%	0.105	1.64(0.90-2.98)	**0.029**	2.06(1.08-3.95)
MRI time	0.078	0.88(0.76-1.01)	-	-

The variables with a p value <0.20 in univariate analysis were included in multivariate logistic regression analysis using a backward likelihood ratio method. No multicollinearity existed among the variables in multivariate regression model. ROC curve analysis were performed for important continuous variables and the optimal cut-off were determined using Youden Index.

* Pure AS represented severe aortic stenosis without more than trace aortic regurgitation; AS = aortic stenosis; MR = mitral regurgitation;
